# Spinal Motion Preservation Surgery

**DOI:** 10.1155/2015/372502

**Published:** 2015-12-30

**Authors:** Jau-Ching Wu, Patrick C. Hsieh, Praveen V. Mummaneni, Michael Y. Wang

**Affiliations:** ^1^Department of Neurosurgery, Taipei Veterans General Hospital, Taipei, Taiwan; ^2^School of Medicine, National Yang-Ming University, Taipei, Taiwan; ^3^Department of Neurosurgery, University of Southern California, Los Angeles, CA, USA; ^4^Department of Neurological Surgery, University of California San Francisco, San Francisco, CA, USA; ^5^Department of Neurosurgery, University of Miami, Coral Gables, FL, USA

The principals of spinal surgery include decompression of neural elements, stabilization of motion segments, and balancing the vertebral alignment. Common operations involve resection of the herniated disc or removal of osteophytes and sometimes stabilization of spondylolisthesis. In extreme cases, for example, correction of scoliosis, fixation of multiple spinal segments and intended bone fusion are often necessary and inevitable, because only arthrodesis can maintain the surgical outcomes for a prolonged time. However, arthrodesis inevitably causes a loss in the range of physiological motion of the spinal segments and consequently may lead to multiple problems, including stiffness, junctional kyphosis, and increased risk of adjacent segment disease (ASD). In the area of spinal surgery during the last decade, much attention therefore has been directed to motion preservation. There are multiple techniques and devices aimed at preserving the segmental range of motion in the treated spine, including disc or facet arthroplasty, laminoplasty, pedicle based dynamic stabilization, and interspinous devices [1–7].

To date, among these innovative surgical approaches to preserve spinal motion, cervical disc arthroplasty (CDA) has the most data on its use and effectiveness. Several multicenter, prospective, randomized, and controlled clinical trials have demonstrated equivalent or superior clinical results of CDA when compared with standard anterior cervical discectomy and fusion (ACDF) [8–11]. Most of the implanted artificial discs have functioned well to maintain a physiological range of motion at the indexed levels [12–14]. However, there has not been enough evidence for the theoretical benefit of CDA on decreasing ASD. Even the true incidence of ASD or its etiologies remain elusive. A study of a cohort of 19,385 patients who underwent ACDF estimated the annual incidence of ASD to be 0.8%, and the accumulative reoperation rate after ACDF was 5.6% in ten years [15]. Although motion preservation surgery of the spine avoids the compensatory increase in the work load at the adjacent levels after spinal arthrodesis surgery, whether ASD can be decreased or postponed by spinal motion preservation surgery remains uncertain. Due to the relatively low incidence rate of ASD, more studies with a larger number of patients and longer term follow-up are required to clarify the outcomes and effects of surgery that preserves spinal motion.

Surgery aiming to preserve motion of the lumbar spine has generated much attention and concern. The lumbar spine inherently endures more weight than the cervical spine and thus makes stabilization as well as preservation of motion even more challenging. Lumbar disc arthroplasty (LDA), in several clinical trials, demonstrated similar improvement in clinical outcomes to spinal fusion surgery [16, 17]. However, there were not sufficient data to support the reduction of ASD after LDA. Moreover, there was less evidence of satisfactory long term outcomes from LDA than has been shown with cervical disc replacement. As there were additional problems related to the approach and difficulty in retrieval, LDA did not gain similar popularity to CDA in the past decade [18].

Others tried pedicle based dynamic stabilization devices since they reportedly provided limited segmental motion and decreased stiffness, but some of these pedicle screws became loose during follow-up [2, 5, 7]. A few studies demonstrated satisfactory clinical outcomes of these dynamic pedicle screws in lumbar degenerative disc disease (DDD) and mild spondylolisthesis [1, 2, 5, 7]. However, the role of lumbar dynamic devices is still debated and is not well accepted.

Another attempt at nonfusion fixation in the lumbar spine was the use of interspinous devices. However, interspinous devices typically led to postoperative focal kyphosis with various degrees of stability provided, depending on its design [19].

The major concerns of these lumbar dynamic devices are the indications for surgery and the durability of the surgery. There are still many debates on the best candidates for such kinds of motion preservation surgery on the lumbar spine. Patients with DDD were the most commonly proposed candidates for these dynamic devices. Nonetheless, it is uncertain if these devices are effective not only for those with DDD but also for others who suffer from disc herniation, hypertrophic ligaments, facet arthropathy, spondylolisthesis, and scoliosis. Such dynamic devices inevitably have wearing problems and lack long term results.

Furthermore, the spinal-pelvic alignment has not been well studied for motion preservation surgery in the lumbar spine. The importance of sagittal balance learned from fusion surgery for deformity has not been translated to or not corroborated for dynamic devices.

In this special issue, there are five papers addressing these state-of-art applications of spinal motion preservation surgery. The papers cover review of material science of CDA, new assessment of three-dimensional movements in lumbar facet joints and vertebrae in patients who underwent LDA, long term (up to almost 9 years) outcomes of cervical laminoplasty in the elderly, comparison of vertebral body stapling and bracing for patients with idiopathic scoliosis, and analysis of lumbar lordosis on screw loosening in Dynesys dynamic stabilization. Although many of these technologies have been on the market for years, more studies are necessary to investigate the long term effects and address the avoidance of associated complications before greater expansion of their utilization.

*Jau-Ching Wu* *Patrick C. Hsieh* *Praveen V. Mummaneni* *Michael Y. Wang*
